# Symptomatic HIV infection and in-hospital outcomes for patients with acute myocardial infarction undergoing percutaneous coronary intervention from national inpatient sample

**DOI:** 10.1038/s41598-024-59920-9

**Published:** 2024-04-29

**Authors:** Mingzhi Cui, Haohong Qi, Ting Zhang, Shixiong Wang, Xiao Zhang, Xiangmei Cao, Xueping Ma, Hui Huang, Ru Yan, Shaobin Jia, Guangzhi Cong

**Affiliations:** 1 Institute of Medical Sciences, General Hospital of Ningxia Medical University, Yinchuan, 750000 Ningxia China; 2 Ningxia University, Yinchuan, 750000 Ningxia China; 3 Vanke School of Public Health, Tsinghua University, Beijing, China

**Keywords:** Cardiovascular diseases, HIV infections

## Abstract

Human immunodeficiency virus (HIV) infection increases the risk of acute myocardial infarction (AMI). However, little is known about its association with in-hospital outcomes and temporal trends in patients with AMI undergoing percutaneous coronary intervention (PCI). We queried patients with AMI who underwent PCI from the National Inpatient Sample Database (2003–2015) and stratified them into three groups: symptomatic, asymptomatic, and HIV-negative. After 1:2 case–control matching (CCM), logistic regression analysis was conducted to determine how HIV infection affected in-hospital outcomes. We also evaluated their recent trends from 2003 to 2015. The total weighted national estimate of 2,191,129 AMI cases included 2,178,995 HIV/AIDS-negative, 4994 asymptomatic, and 7140 symptomatic HIV cases. Symptomatic but not asymptomatic patients with HIV suffered more than triple the in-hospital mortality (adjusted odds ratio (aOR) 3.6, 95% confidence interval (CI) 2.5–5.2), over one-fold incidence of acute kidney injury (aOR 2.6 95% CI 1.9–3.4) and cardiogenic shock risk (aOR 1.9, 95% CI 1.3–2.7), a longer length of hospital stay (beta 1.2, 95% CI 1.0–1.5), and had more procedures (beta 1.3, 95% CI 1.2–1.5). These disparities relating to symptomatic HIV infection persisted from 2003 to 2015. In patients with AMI who underwent PCI, symptomatic HIV infection was associated with higher in-hospital mortality and more severe outcomes.

To date, the human immunodeficiency virus (HIV) has resulted in 40.1 million fatalities worldwide, with an estimated range of 33.6 to 48.6 million^1^. With early and influential antiretroviral therapy, HIV transforms from a life-threatening condition to a manageable, chronic illness with an elevated risk of accompanying chronic disorders, mainly cardiovascular diseases (CVDs)^2^. Over the past two decades, the incidence of HIV-related CVDs has increased, resulting in 2.6 million disability-adjusted years annually^3^. Recent studies have shown a doubling number of patients with HIV who experience acute myocardial infarction (AMI)^4^. Although no clear guidelines exist, there is evidence of treatment bias in patients with HIV hospitalized for AMI^5^. Timely percutaneous coronary intervention (PCI) is currently the recommended reperfusion therapy for AMI^5^. However, a cohort study found that HIV was associated with increased in-hospital mortality in patients with HIV who underwent PCI^6^. Despite these findings, the exact impact of HIV on in-hospital outcomes and temporal trends in patients with HIV undergoing PCI remains unknown.

Although there is a growing focus on reducing the risk of coronary heart disease and promoting primary prevention in patients with HIV, literature on the clinical treatments and outcomes of AMI among these patients remains limited. Clinical trials on AMI have not included patients with HIV, and observational studies have not differentiated between symptomatic and asymptomatic patients with HIV. However, early HIV infection can progress to persistent asymptomatic illness or clinical acquired immunodeficiency syndrome (AIDS)^7^.

Consequently, recommendations based on published clinical studies on AMI may not be directly relevant to HIV-infected individuals. Additionally, the rarity of AIDS makes it difficult to conduct large-scale studies assessing the influence of HIV on PCI outcomes^8^.

We have previously reported that symptomatic HIV infection was associated with worse in-hospital outcomes in patients with a pre-existing peripheral arterial disease requiring vascular intervention^9^. Therefore, in this study, we sought to investigate the impact of symptomatic HIV infection on in-hospital outcomes and temporal trends among patients undergoing PCI using the nationally representative National Inpatient Sample (NIS) from 2003 to 2015.

## Methods

### Data source

With a national annual weighted estimate of more than 35 million admissions, the NIS database, which was used in this study, is the largest openly accessible inpatient healthcare database in the USA. The NIS collects data from over 1000 participating hospitals on approximately 8 million inpatient stays annually, and includes patient demographics such as age, sex, and race, as well as information on pre-existing conditions, procedures performed, and primary and secondary discharge diagnoses. It has been applied and validated in multiple research studies reporting disease trends and hospital outcomes of medical conditions^10^.

### Study population

**Figure 1:**
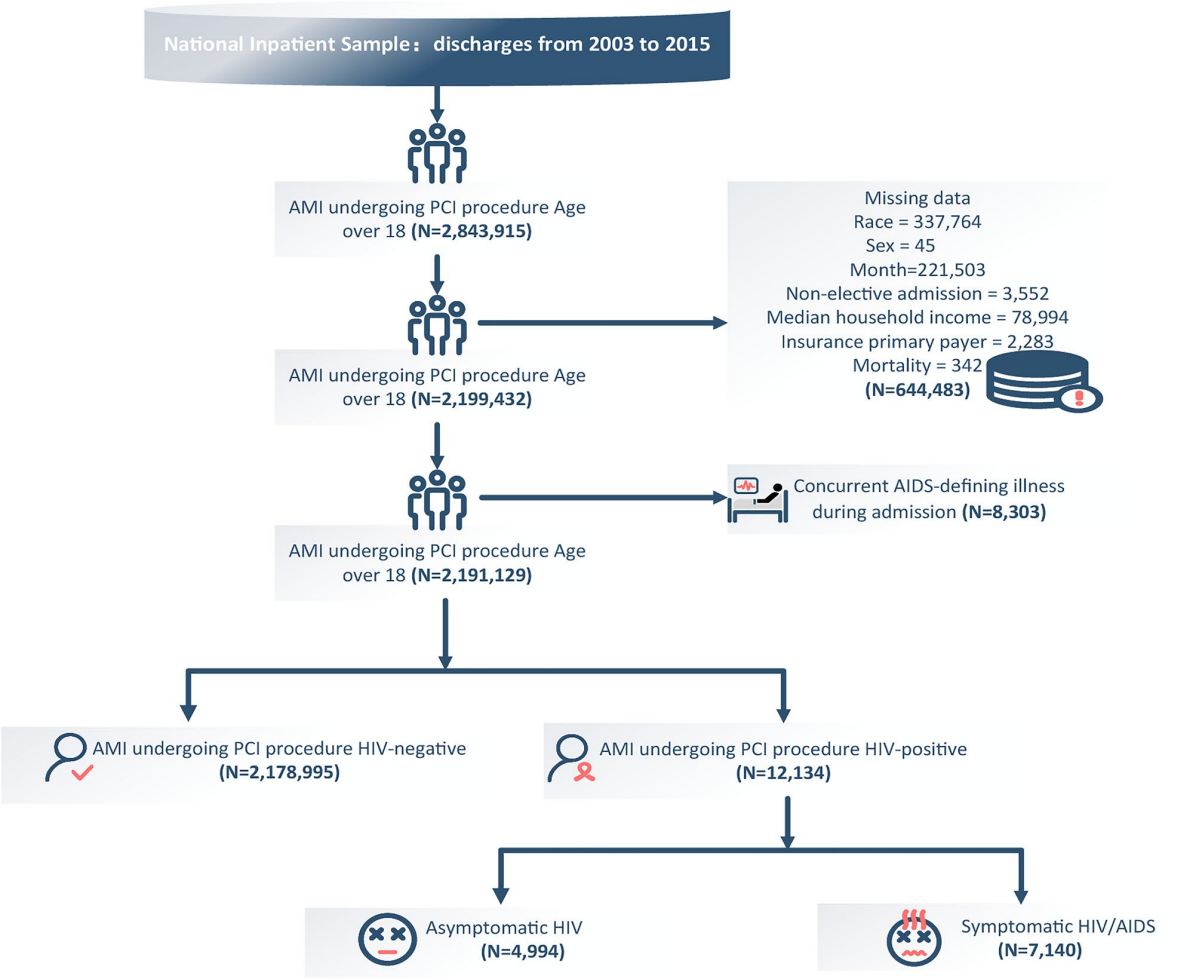
Flow diagram of study population selection. Weighted selection of 2,843,915 patients with AMI undergoing PCI aged over 18 from the National Inpatient Sample database. A total of 2,191,129 patients were selected after excluding missing data and HIV complications. The selected patients were separated into an HIV-negative group of 2,178,995 patients, and an HIV-positive group of 12,134 patients. The HIV-positive group was then separated into a symptomatic HIV/AIDS group of 7,140 patients and an asymptomatic HIV/AIDS group of 4,994 patients.

We used the International Classification of Diseases-9th Revision Clinical Classification Software code (100) to search the NIS database from 2003 to 2015 to identify hospitalizations for AMI. We selected patients with a primary diagnosis of AMI undergoing PCI (International Classification of Diseases, Ninth Revision, Clinical Modification [ICD-9-CM] code 410, [ICD-9-PCS] code 00.66, 36.06 and 36.07) and a secondary diagnosis of HIV (ICD-9-CM codes V08 and 042)^11–13^. According to the WHO, the four clinical stages of HIV infection are acute HIV infection, asymptomatic HIV infection, symptomatic HIV infection, and AIDS^14^. This diagnosis can be accurately identified using the ICD code^15^. Overall, we identified the weighted records of 2,191,129 patients with AMI who underwent PCI procedures after excluding records of patients younger than 18 or with missing data or concurrent AIDS-defining diseases during admission. The cohort was further classified as symptomatic HIV, asymptomatic HIV, and HIV-negative. A flowchart of this study is shown in Fig. 1, and Supplementary Table S1 lists the ICD-9 codes employed.

### Variable outcomes

The primary outcome of this study was in-hospital mortality rate. Secondary outcomes included bleeding, cardiogenic shock (CS), acute kidney injury (AKI), length of stay, and the number of procedures during hospitalization.

### Statistical analysis

All data processing and analyses were carried out according to the NIS database documentation and methodological criteria, which consider the complicated survey design of clustering and stratification and the possibility of sampling bias. Categorical variables are presented as numbers (percentages) and were compared using the chi-square test, while continuous variables were compared using the Student’s t-test and are reported as mean or median (interquartile range [IQR]) depending on whether they were normally distributed or not. These were used to compare demographic and comorbidities among patients with AMI, with asymptomatic HIV infection, symptomatic HIV disease/AIDS, and HIV-uninfected.

To reduce bias from the observed covariates, we used a 1:2 CCM between the HIV-infected and uninfected control groups to construct matched cohorts based on age, sex, smoking status, and comorbidities. Multivariate regression analysis was conducted among patients with asymptomatic HIV, symptomatic HIV disease/AIDS, and those without HIV. The model included the following independent variables: age, sex, race, expected primary payment, median household income, smoking status, prior coronary artery bypass graft (CABG), AMI types, and comorbidities. In addition, the temporal dynamics of in-hospital outcomes were compared among the three groups.

The statistical software package R, version 4.2.0 (available at: http://www.R-project. org; R Foundation for Statistical Computing, Vienna, Austria), and Empower-Stats 4.1 (X&Y Solutions, Inc, Boston, Mass, USA) were used for all statistical analysis. All pooled estimates were presented with 95% confidence intervals (CI), and alpha criteria (*p* < 0.05) was regarded as statistically significant.

### Ethical approval

As the database contains deidentified patient information, the study was deemed exempt from the need for Institutional Review Board approval.

## Results

### Study population

Of the 2,191,129 patients with AMI who met all the inclusion and exclusion criteria, 12,134 (0.6%) were diagnosed with HIV infection. Among them, 7,140 (58.8%) were HIV-positive but had no history of HIV-related complications, classifying them as having asymptomatic HIV infection. The ICD-9-CM documented the remaining 4,994 patients (41.2%) with symptomatic HIV or AIDS according to the CDC clinical disease classification. The baseline characteristics of the study population before and after 1:2 case–control matching (CCM) are shown in Table 1 and Supplementary Table S4 respectively. Among the three cohorts, the types of acute myocardial infarction were consistently distributed with the majority being non-ST-segment elevation myocardial infarction (Table 1). We also explored the crude association between different HIV statuses and baseline demographic and clinical characteristics (Supplementary Table S2). Overall, the results of the univariate analyses were consistent with those presented in Table 1.

**Table 1:** Baseline characteristics of patients with AMI who underwent PCI, grouped by HIV status.

Variable	HIV-negative (n = 2,178,995)	Asymptomatic HIV (n = 4,994)	Symptomatic HIV (n = 7,140)	p-value*
Age (years) (survey-weighted mean (95% CI))	63.7 (63.2–64.1)	54.1 (51.4–56.8)	69.3 (67.1–71.5)	< 0.001
Female (%)	33.2 (32.2–34.1)	11.4 (7.1–17.8)	32.6 (23.4–43.4)	< 0.001
Race (%)				< 0.001
Caucasian	79.1 (76.6–81.5)	35.9 (22.2–52.4)	87.8 (84.2–90.7)	
African American	7.0 (6.1–7.9)	25.0 (15.6–37.7)	5.5 (4.0–7.6)	
Hispanic	8.2 (6.2–10.9)	36.0 (14.7–64.7)	3.7 (2.6–5.3)	
Asian or Pacific Islander	1.7 (1.4–2.1)	0.3 (0.1–1.0)	0.8 (0.4–1.5)	
Native American	0.9 (0.5–1.6)	0.10 (0.0–0.7)	0.2 (0.1–0.7)	
Other	3.1 (2.6–3.7)	2.7 (1.4–4.9)	1.9 (1.2–3.0)	
Primary expected payer (%)				0.004
Medicare	42.5 (39.8–45.3)	49.3 (29.2–69.6)	51.0 (39.7–62.1)	
Medicaid	7.3 (6.3–8.5)	11.3 (7.0–17.7)	4.1 (2.9–5.7)	
Private insurance	31.2 (29.5–32.9)	19.9 (12.6–29.9)	15.9 (12.3–20.3)	
Other	19.0 (16.1–22.4)	19.6 (12.3–29.6)	29.1 (16.9–45.3)	
Median household income (%)				0.274
1st quartile	29.0 (25.5–32.8)	42.4 (23.9–63.2)	23.4 (14.8–34.9)	
2nd quartile	26.3 (23.8–29.0)	16.1 (10.1–24.7)	23.2 (15.5–33.1)	
3rd quartile	22.1 (19.8–24.6)	14.1 (8.9–21.6)	24.1 (15.6–35.4)	
4th quartile	22.6 (19.5–26.0)	27.5 (9.8–56.8)	29.3 (20.3–40.3)	
Smoking (%)	42.2 (39.7, 44.7)	54.7 (33.4, 74.4)	37.5 (27.9–48.3)	0. 320
Comorbidity (%)				
Previous coronary artery bypass graft	4.6 (4.1–5.2)	1.9 (1.0–3.5)	7.9 (2.5–22.5)	0.346
Hypertension	58.4 (57.6–59.2)	52.2 (31.9–71.8)	61.5 (52.4–69.8)	0.692
Hyperlipidemia	53.9 (51.4–56.3)	64.1 (47.9–77.6)	40.6 (30.6–51.4)	0.029
Diabetes mellitus	31.4 (30.1–32.7)	30.9 (12.7–57.9)	26.3 (18.4–36.2)	0.825
Congestive heart failure	17.6 (16.5–18.8)	9.1 (5.6–14.4)	24.6 (16.0–35.8)	0.021
Chronic obstructive pulmonary disease	8.7 (8.2–9.1)	5.2 (3.1–8.5)	30.3 (18.1–46.1)	< 0.001
Weight loss	0.1 (0.1–0.2)	0.2 (0.0–0.8)	0.7 (0.3–1.4)	< 0.001
Chronic kidney disease	10.0 (8.5–11.7)	8.1 (5.0–12.8)	10.6 (8.1–13.8)	0.607
Ischemic stroke	3.5 (2.8–4.4)	2.2 (1.2–3.9)	8.6 (2.4–26.6)	0.143
AMI type (%)				0.241
ST-segment elevation myocardial infarction	42.6 (40.6–44.7)	31.8 (19.8–46.8)	47.5 (36.5–58.75)	
Non–ST-segment elevation myocardial infarction	57.4 (55.3–59.4)	68.2 (53.2–80.2)	52.5 (41.3–63.5)	

*For continuous variables: survey-weighted mean (95% CI); the p-value was determined by survey-weighted linear regression (svyglm).

For categorical variables: survey-weighted percentage (95% CI) and the p-value were calculated using the survey-weighted chi-square test (svytable).

### Primary and secondary outcomes

As shown in Table 2, the symptomatic HIV group had significantly higher in-hospital mortality (5.6%), followed by the no HIV group (2.1%) and the asymptomatic HIV group (0.7%, *p* < 0.0001). Additionally, patients with AMI in the symptomatic HIV infection group had the highest risk of CS (4.5%), AKI (10.1%), extended hospital stays, and more procedures.

**Table 2:** Outcomes of patients with AMI who underwent PCI, grouped by HIV infection status.

Outcomes	HIV-negative (n = 2,178,995)	Asymptomatic HIV (n = 4,994)	Symptomatic HIV (n = 7,140)	p-value*
In-hospital mortality (%)	2.1 (1.8–2.4)	0.7 (0.3–1.6)	5.6 (4.1–7.6)	< 0.001
Complications (%)				
Cardiogenic shock	3.4 (3.1–3.8)	1.6 (0.8–3.0)	4.5 (3.2–6.2)	0.017
Bleeding	3.7 (2.7–5.0)	2.3 (1.3–4.1)	3.3 (2.3–4.8)	0.337
Acute kidney injury	7.9 (7.1–8.8)	5.1 (3.1–8.3)	10.1 (7.7–13.1)	0.066
Length of hospital stay (d)	3.6 (3.5–3.8)	2.8 (2.4–3.3)	5.3 (4.9–5.8)	< 0.001
Number of ICD-9-CM procedures on this discharge	6.9 (6.6–7.2)	6.9 (6.4–7.4)	7.4 (7.1–7.6)	0.038

*For continuous variables: survey-weighted mean (95% CI); the p-value was determined by survey-weighted linear regression (svyglm).

For categorical variables, survey-weighted percentage (95% CI) and the p-value were calculated using the survey-weighted chi-square test (svytable).

**Figure 2:**
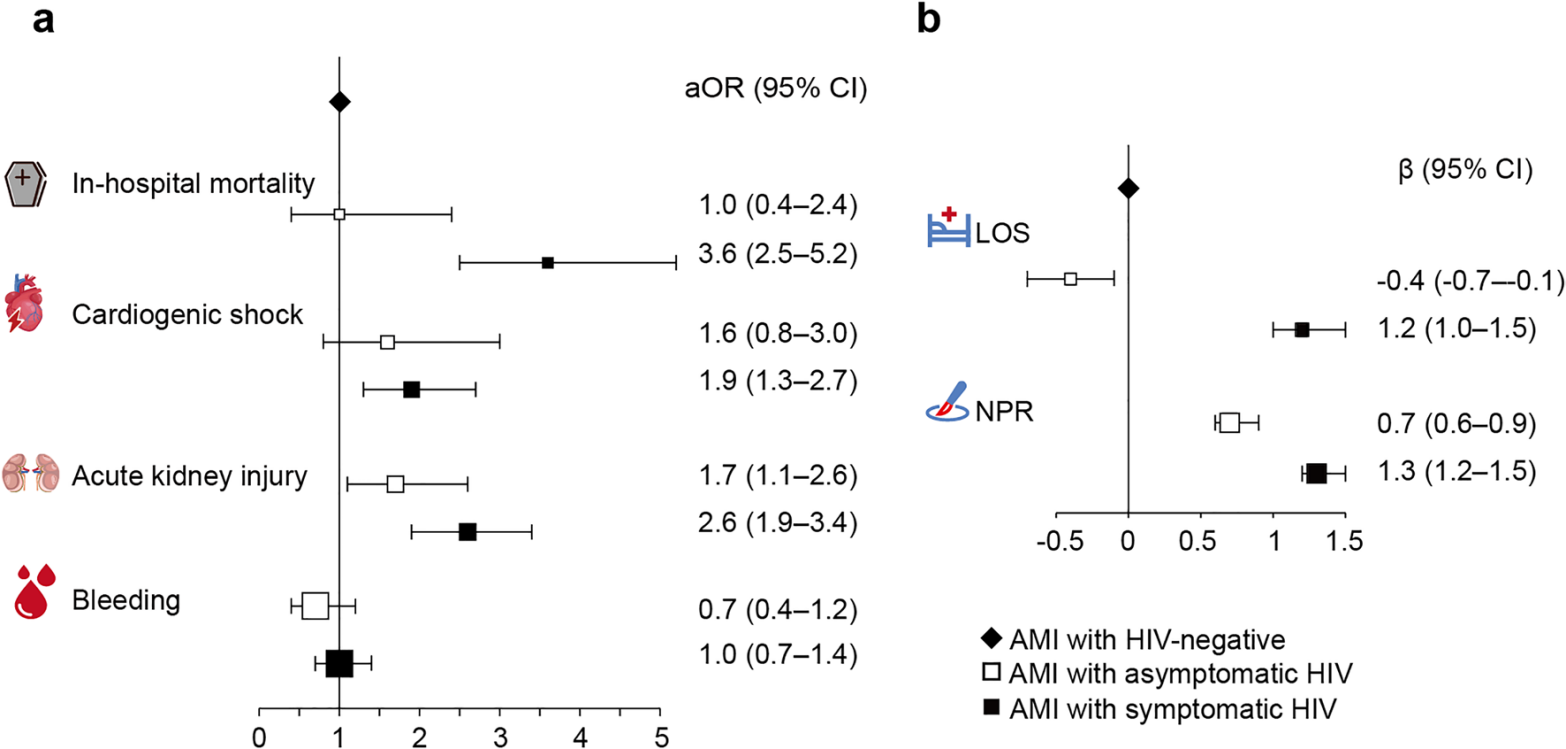
In-hospital mortality and adverse outcomes in 1:2 case–control matching model. (**a**) Adjusted odd ratios for in-hospital mortality and outcomes. (**b**)Adjusted *β* value for length of stay (LOS) and additional procedures during hospitalization (NPR); represented as odds ratio and *β* value (95% CI). Adjusted for age, sex, race, primary payer, socioeconomic status, smoking, prior coronary artery bypass graft, types of AMI, and comorbidities.

After 1:2 CCM, adjusting for demographics and comorbidities (Fig. 2), the group of patients with symptomatic HIV/AIDS had a 3.6-fold higher risk of in-hospital mortality compared to HIV-negative patients (aOR 3.6, 95% CI 2.5–5.6), while the asymptomatic HIV group had no significant risk for in-hospital mortality (aOR 1.0, 95% CI 0.4–2.4). Furthermore, symptomatic HIV/AIDS was an independent risk factor for acute kidney failure (aOR 2.6, 95% CI 1.9–3.4) and CS (aOR 1.9, 95% CI 1.3–2.7), whereas asymptomatic HIV infection was not. Moreover, the patients with symptomatic HIV/AIDS had longer length of stay (β 1.2, 95% CI 1.0–1.5) and underwent more than one additional procedure (β 1.30, 95% CI 1.2–1.5) than the HIV-negative patients. Similar tendencies were observed in the non-matching multiple regression equations model (Supplementary Fig. S1). In addition, we found that symptomatic HIV/AIDS was associated with adverse outcomes during hospital stay in different subgroups. (Supplementary Table S3).

### Temporal trends of in-hospital outcomes by HIV infection

**Figure 3:**
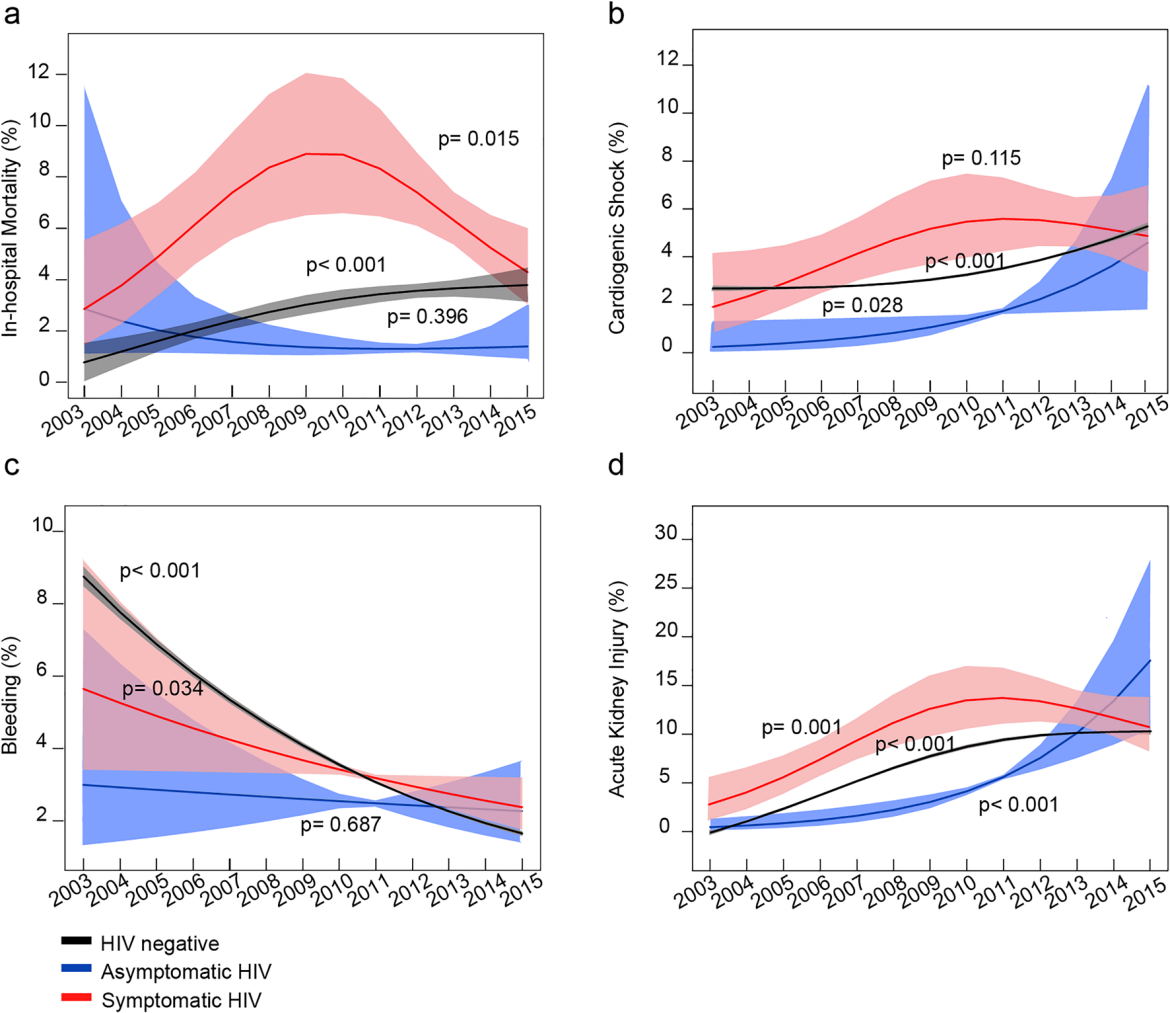
Temporal trend of in-hospital mortality and outcomes. In the symptomatic HIV infection group, there was a fluctuating trend in the risk of in-hospital mortality. However, the risk was still higher than that of the HIV/AIDS-negative group and the asymptomatic HIV/AIDS group. (**a**) The in-hospital mortality (symptomatic patients with HIV/AIDS:1. 7% in 2003, 15.5% in 2008, compared with 8.6% in 2015) (**b**) The cardiogenic shock rate (symptomatic patients with HIV/AIDS: 0.8% and 6.6% in 2003–2015) is increasing over time and higher than the HIV/AIDS-negative group and the asymptomatic HIV/AIDS group. (**c**) The bleeding risk had decreased (symptomatic patients with HIV/AIDS: 5.8% and 3.3% in 2003–2015), and the overall trend is similar to the HIV/AIDS-negative and asymptomatic HIV/AIDS group. (**d**)The acute renal injury rate had a steady increase (symptomatic patients with HIV/AIDS: 4.2% and 16.6% in 2003–2015) and higher than the HIV/AIDS-negative group and the asymptomatic HIV/AIDS group.

From 2003–2015, the prevalence of in-hospital mortality in symptomatic patients with HIV/AIDS patients ranged from 1.7% to 15.5%, peaking in 2008. Moreover, it was consistently higher than that in the HIV-negative and asymptomatic HIV groups (Fig. 3a). The prevalence of CS increased from 0.8% in 2003 to 6.6% in 2015 in patients with symptomatic HIV/AIDS, and this was consistently higher than in the asymptomatic HIV and HIV-negative groups (Fig. 3b). There was a downward trend in bleeding in patients with AMI who underwent PCI between 2003 and 2015, with no significantly differences between the three groups (Fig. 3c). The risk of renal injury in patients with symptomatic HIV/AIDS who underwent PCI increased from 4.2% in 2003 to 16.6% in 2015, and this was higher than in the asymptomatic HIV and HIV-negative groups (Fig. 3d).

## Discussion

In this study, we evaluated the effects of the HIV infection status on in-hospital outcomes and temporal trends between 2003 and 2015 in patients with AMI who underwent PCI in a nationally representative population. Our significant findings are as follows: (1) patients with symptomatic HIV infection have a higher in-hospital mortality than the HIV-negative cohort; and (2) symptomatic HIV infection was also associated with risk of AKI, CS, longer length of stay, and additional procedures during hospitalization. Our study sheds light on the impact of HIV infection on in-hospital outcomes of patients with AMI who underwent PCI, an area that has not been extensively studied. This study provides evidence that patients with symptomatic HIV infection are at a higher risk of mortality and other adverse outcomes during hospitalization, which can help inform clinical decision-making and improve patient outcomes. This study also contributes to the field by highlighting the importance of considering the HIV status when managing patients with AMI who undergo PCI and underscores the need for more research in this area.

Firstly, our results suggest that patients with symptomatic HIV infection are at a higher risk of in-hospital mortality during AMI hospitalization than asymptomatic HIV infection, even though they underwent PCI. Several studies have reported- that HIV-infected patients have an increased risk of developing CVDs and related outcomes, including AMI. HIV infection is associated with chronic inflammation and immune activation, which contribute to the development of CVDs. Furthermore, Singh et al.^16^ reported that HIV-infected patients with AMI have a higher in-hospital mortality risk. These patients were less likely to undergo revascularization procedures, such as coronary angiography and PCI, than non-HIV-infected patients with AMI. Another study indicated that treatment bias or difference in in-hospital mortality for HIV-seropositive patients presenting with AMI compared to the general population did not exist based on an analysis of the NIS database from 2010 to 2014^17^. However, the study did not distinguish patients with AMI according to their HIV infection status (symptomatic or asymptomatic), which may have contributed to this controversy. Recently, it was reported that despite well-controlled HIV infection, better overall cardiovascular risk profile, and similar PCI procedural metrics, patients with HIV infection still have significantly worse long-term survival following PCI than controls^18^. As shown in Table 1, patients with symptomatic HIV infection are different from patients with asymptomatic HIV infection, being older, more likely to be female, having fewer traditional cardiovascular risk factors, but more comorbidities such as heart failure and chronic obstructive pulmonary disease. In addition, the challenges and barriers to HIV care engagement such as access to healthcare, stigma and vulnerability and mental health issues may play a role for the outcomes dispirited between symptomatic and asymptomatic HIV infection^19^.

To account for these potential confounders, we distinguished patients according to their HIV infection status and performed a 1:2 CCM and multiple variable logistic regression. Our results suggest that symptomatic HIV infections are associated with higher in-hospital mortality, even in patients who underwent PCI. Interestingly, as shown in Table 1, the population with asymptomatic HIV is much younger and less likely to have comorbidities such as congestive heart failure and chronic obstructive pulmonary disease than HIV-negative individuals. This age and comorbidity difference may explain the lower in-hospital mortality in asymptomatic patients than in the HIV-negative population.

In addition to in-hospital mortality, another important finding of this study was that symptomatic patients are at higher risk of adverse in-hospital outcomes, including CS, AKI, extended hospital stay, and more procedures during the AMI hospitalization. CS^20^, AKI^21^, and bleeding^22^ represent the most common and fatal complications associated with PCI. As shown in Table 2, the prevalence of CS, AKI, and bleeding was 4.5% (95% CI 3.2–6.2), 10.1% (95% CI 7.7–13.1), and 3.3% (95% CI 2.3–4.8) respectively in patients with symptomatic HIV infection. For these patients, the risks of CS and AKI were much higher than for patients with asymptomatic HIV infection. Because of the low prevalence of HIV infection, the relationship between HIV infection, CS, AKI, bleeding, extended hospital stays, and additional procedures has rarely been explored. According to the Mechanical Complications of Acute Myocardial Infarction: A Scientific Statement From the American Heart Association^20^, the risk factors for CS following AMI include older age, female sex, history of heart failure, and chronic kidney disease. In our study, the higher proportion of females and prevalence of AKI in patients with symptomatic HIV infection may have contributed to the higher risk of CS, resulting in higher in-hospital mortality and extended hospital stay. Furthermore, chronic kidney insufficiency in the symptomatic cases occurs in 3.5–48.5%^23^, making patients with symptoms more vulnerable to AKI. Our results suggest that when confronted with AMI and a history of symptomatic HIV infection, more attention should be paid to monitoring their risk of CS and AKI.

A meta-analysis^24^ of six studies conducted between 2003 and 2015, including 2268 patients, suggested that after PCI, patients with HIV had similar mortality and major adverse cardiac events to those without HIV infection. However, we noticed a significant gap in the in-hospital outcomes between symptomatic and HIV-negative patients. Our results indicate that in-hospital outcomes disparities related to HIV infection in patients with AMI who underwent PCI persisted from 2003 to 2015. We assume that the limited sample size and potential confounders owing to asymptomatic HIV infection in the meta-analysis may have contributed to this difference. For HIV-negative patients with AMI who underwent PCI, the in-hospital mortality increased slightly annually, which is consistent with three contemporary studies from the USA^25^, Canada^26^, and Sweden^27^. Nevertheless, for symptomatic HIV patients, as shown in Fig. 3, in-hospital mortality and other complications were still worse than for HIV-negative patients and for those with asymptomatic HIV infection, even when PCI was performed. Interestingly, the in-hospital mortality from symptomatic HIV infection with AMI who underwent PCI sharply declined from approximately 8% in 2008 to 4% in 2015, which may have originated from the broad application of new-generation drug-eluting stents^16^ and the progress of antiviral therapy. Despite the impact of symptomatic HIV infection on the clinical outcomes of AMI, a Scientific Statement From the American Heart Association^28^ for people living with HIV provides limited recommendations for evaluating and managing HIV patients during heart attacks. Patients with symptomatic HIV infection are particularly vulnerable to acute myocardial infarction. Therefore, extra attention should be paid to the perioperative management of these vulnerable patients to reduce in-hospital mortality and adverse events.

## Strengths and limitations

Our study exploring the effect of HIV infection on patients with AMI undergoing PCI, based on the NIS database, has the largest sample size to date. Based on the stages of HIV infection, patients with AMI were divided into symptomatic and asymptomatic groups to, identify the most vulnerable patients. Furthermore, we excluded patients with HIV complications, including Kaposi’s sarcoma, candidiasis, cytomegalovirus, and cryptococcosis, and adopted a 1:2 CCM model to adjust for potential bias, which made our results more reliable. However, our study has some limitations. The NIS is a management database prone to coding and documentation errors. As a time-discrete database related to certain hospitalizations, patients cannot be followed-up for a long time. In addition, some clinical information, including operation details, nursing information, laboratory and imaging results, medications, coronary stent types, and antiviral therapy that may influence in-hospital outcomes were not available.

## Conclusion

**Figure 4:**
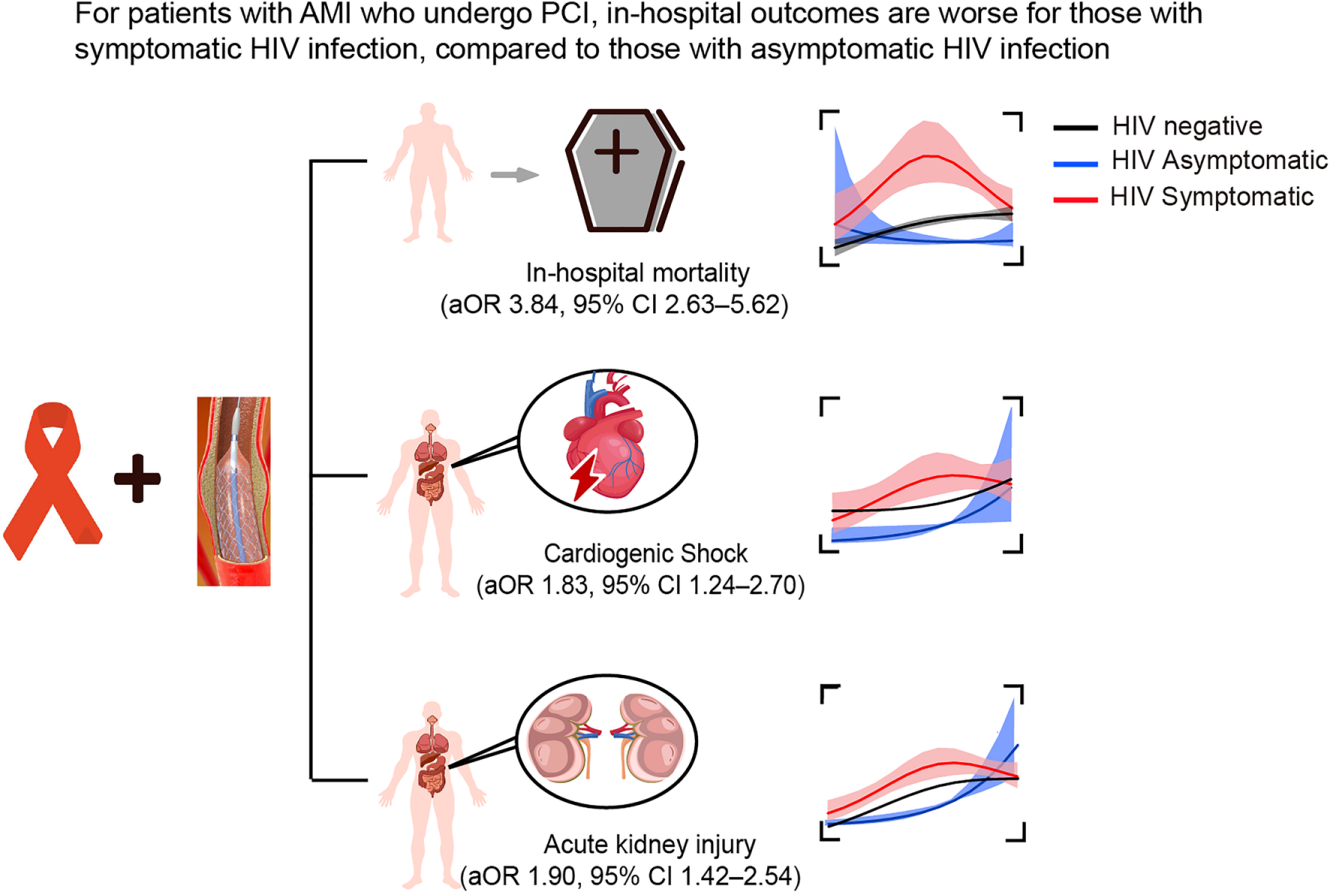
Graphic of study findings. For patients with AMI who underwent PCI, patients with symptomatic HIV/AIDS have higher in-hospital mortality and rates of cardiac shock and acute kidney injury.

In patients with AMI who underwent PCI, symptomatic HIV infection was associated with higher in-hospital mortality, AKI, CS, extended hospital stay, and more procedures during hospitalization (Fig. 4). These disparities related to symptomatic HIV infection persisted from 2003 to 2015. More attention should be paid to symptomatic HIV patients with comorbid AMI undergoing PCI procedures.

### Supplementary Information

Supplementary Information.

## Data Availability

The data supporting the findings of this study are available in the NIS database. The NIS database was purchased by the researchers. The data sources are publicly accessible (https://cdors.ahrq.gov/databases). The raw data supporting the conclusions of this article will be made available by the authors without any reservations. All other supporting data in this study are available from Mingzhi Cui, MD and Guangzhi Cong, PhD upon request.
