# Supplementary material for: Symptomatic HIV infection and in-hospital outcomes for patients with acute myocardial infarction undergoing percutaneous coronary intervention from national inpatient sample
Source: Sci Rep. 2024 Apr 29;14:9832. doi: 10.1038/s41598-024-59920-9 (PMC11058195; doi:10.1038/s41598-024-59920-9)
Supplement: Supplementary file 1 — Supplementary Information. [file 41598_2024_59920_MOESM1_ESM.pdf]

Symptomatic HIV infection and in-hospital outcomes for patients with acute myocardial infarction undergoing percutaneous coronary intervention: an insight from a national inpatients sample

Mingzhi Cui, MD[1]†, Haohong Qi, MD [1]†, Ting Zhang, MD [2], Shixiong Wang, MD [1], Xiao Zhang, Ph.D. [3], Xiangmei Cao, MD. Ph.D. [1], Xueping Ma[1], Hui Huang[1], Ru Yan, MD[1]*, Shaobin Jia, Ph.D.[1]*, and Guangzhi Cong, Ph.D.[1]*
1Institute of Medical Sciences, General Hospital of Ningxia Medical University, Yinchuan, Ningxia, 750000, China
2 Ningxia University, Yinchuan, Ningxia, 750000, China
3Vanke School of Public Health, Tsinghua University, Beijing, China
† These authors contributed equally to this work and share first authorship.
*** Correspondence:**
Corresponding Author: Ru Yan, Shaobin Jia, and Guangzhi Cong.

Supplementary Table S1 International Classification of Diseases, Ninth Edition, Clinical Modification (ICD-9-CM) codes used to identify the baseline comorbidities, procedures, and in-hospital outcomes

**Variable**	**Codes**
**Study population**	
**HIV status**	
**Asymptomatic HIV**	V08
**Symptomatic HIV**	042
**Smoking**	305.1–V15.82
**Comorbidity**	
**Previous Coronary Artery Bypass Graft**	V45.81
**Hypertension**	401.0–401.1–401.9
**Hyperlipidemia**	272.4
**Diabetes Mellitus**	250.00–99
**Congestive Heart Failure**	428.20-49
**Chronic Obstructive Pulmonary Disease**	496
**Weight Loss**	783.21
**Chronic Kidney Disease**	585.1–9
**Ischemic Stroke**	V1254
**AMI type**	
**ST-segment Elevation Myocardial Infarction**	410.0x–410.1x–410.2x–410.3x–410.4x–410.5x–410.6x–410.8x–410.9x
**Non–ST-segment Elevation Myocardial Infarction**	410.7x
**Outcomes**	
**Cardiogenic Shock**	785.51
**Bleeding**	998.11–998.12
**Acute Kidney Injury**	584.5-9
**Concurrent AIDS-defining illness**	
**Candidiasis**	112.x
**Kaposi Sarcoma**	176.x
**Cytomegalovirus Infection**	078.5

**Cryptococcosis**	117.5
**Procedures**	
**Percutaneous Transluminal Coronary Angioplasty**	00.66
**Single vessel percutaneous transluminal coronary angioplasty or coronary atherectomy without mention of thrombolytic agent**	36.01
**Single vessel percutaneous transluminal coronary angioplasty or coronary atherectomy with mention of thrombolytic agent**	36.02
**Multiple vessel percutaneous transluminal coronary angioplasty or coronary atherectomy performed during the same operation–with or without mention of thrombolytic agent**	36.05
**Insertion of non-drug-eluting coronary artery stent(s)**	36.06
**Insertion of drug-eluting coronary artery stent(s)**	36.07
**Left heart cardiac catheterization**	37.22
**Combined right and left heart cardiac catheterization**	37.23
**Implant of pulsation balloon**	37.61
**Other and unspecified coronary arteriography**	88.57
**Coronary arteriography using two catheters**	88.56
**Angiocardiography of left heart structures**	88.53

Supplementary Table S2 Univariate analysis of the risk factors of in-hospital mortality and outcomes in AMI patients undergoing PCI grouped by HIV status

Characteristic	**In-hospital mortality** OR, (95% CI) *P*-value	**Acute Kidney Injury** OR, (95% CI) *P*-value	**Cardiogenic shock** OR, (95% CI) *P*-value	**Bleeding** OR, (95% CI) *P*-value	**Length of Hospital Stay** *β*, (95% CI) *P*-value	**Number of ICD-9-CM Procedures on this discharge** *β*, (95% CI) *P*-value
**HIV status**						
**HIV-negative**	1.0	1.0	1.0	1.0	0	0
**Asymptomatic HIV**	0.3 (0.2–0.7) 0.004	0.6 (0.5–0.8) 0.001	0.5 (0.3–0.8) 0.002	0.6 (0.4–0.9) 0.022	-0.8 (-1.0–-0.6) <0.001	-0.1 (-0.2–0.1) 0.361
**Symptomatic HIV**	2.8 (2.3–3.5) <0.001	1.3 (1.1–1.5) 0.003	1.3 (1.0–1.7) 0.035	0.9 (0.7–1.2) 0.406	1.7 (1.5–1.9) <0.001	0.4 (0.3–0.6) <0.001
**Age (Survey-weighted mean (95% CI))**	1.1 (1.1–1.1) <0.001	1.0 (1.0–1.0) <0.001	1.0 (1.0–1.0) <0.001	1.0 (1.0–1.0) <0.001	0.0 (0.0–0.0) <0.001	0.0 (0.0–0.0) <0.001
**Female (%)**	1.7 (1.7–1.8) <0.001	1.5 (1.4–1.5) <0.001	1.3 (1.3–1.4) <0.001	1.7 (1.6–1.7) <0.001	0.7 (0.7–0.7) <0.001	-0.0 (-0.0–-0.0) 0.001
**RACE (%)**						
**Caucasian**	1.0	1.0	1.0	1.0	0	0
**African American**	0.6 (0.6–0.7) <0.001	1.5 (1.5–1.6) <0.001	0.6 (0.6–0.7) <0.001	0.8 (0.7–0.8) <0.001	0.3 (0.2–0.3) <0.001	-0.0 (-0.1–-0.0) <0.001
**Other**	1.0 (0.9–1.0) 0.814	1.2 (1.2–1.2) <0.001	1.0 (0.9–1.0) 0.155	1.1 (1.0–1.1) 0.002	0.3 (0.3–0.3) <0.001	0.3 (0.3–0.3) <0.001
**Primary expected payer (%)**						
**Medicare**	1.0	1.0	1.0	1.0	0	0
**Medicaid**	0.4 (0.4–0.4) <0.001	0.6 (0.6–0.6) <0.001	1.3 (1.3–1.4) <0.001	0.8 (0.8–0.9) <0.001	-0.2 (-0.2–-0.1) <0.001	0.3 (0.2–0.3) <0.001
**Private Insurance**	0.3 (0.3–0.3) <0.001	0.3 (0.3–0.3) <0.001	0.6 (0.6–0.6) <0.001	0.9 (0.9–0.9) <0.001	-1.2 (-1.2–-1.1) <0.001	-0.0 (-0.1–-0.0) <0.001
**Other**	0.5 (0.5–0.6) <0.001	0.7 (0.6–0.7) <0.001	1.1 (1.1–1.2) <0.001	0.5 (0.5–0.6) <0.001	-1.0 (-1.1–-1.0) <0.001	0.0 (0.0–0.1) 0.009
**Median household income (%)**						
**1st quartile**	1.0	1.0	1.0	1.0	0	0
**2nd quartile**	0.9 (0.9–1.0) 0.005	1.0 (1.0–1.0) 0.199	1.0 (1.0–1.1) 0.472	1.0 (1.0–1.0) 0.809	-0.1 (-0.2–-0.1) <0.001	-0.1 (-0.1–-0.0) <0.001
**3rd quartile**	0.7 (0.7–0.8) <0.001	0.9 (0.9–1.0) <0.001	1.1 (1.1–1.2) <0.001	1.0 (1.0–1.1) 0.221	-0.2 (-0.2–-0.1) <0.001	0.1 (0.1–0.1) <0.001
**4th quartile**	0.8 (0.8–0.9) <0.001	0.7 (0.7–0.8) <0.001	1.0 (0.9–1.0) 0.486	0.9 (0.9–1.0) <0.001	-0.3 (-0.3–-0.3) <0.001	0.2 (0.2–0.2) <0.001
**Smoking (%)**	0.4 (0.4–0.4) <0.001	0.5 (0.4–0.5) <0.001	0.8 (0.8–0.8) <0.001	0.7(0.6–0.7) <0.001	-0.8 (-0.8–-0.8) <0.001	-0.04 (-0.06–-0.03) <0.001
**Comorbidity (%)**						
**Previous Coronary Artery Bypass Graft**	0.6 (0.5–0.6) <0.001	1.5 (1.4–1.5) <0.001	0.4 (0.4–0.5) <0.001	0.5 (0.4–0.5) <0.001	0.1 (0.0–0.1) <0.001	-0.2 (-0.2–-0.2) <0.001

**Hypertension**	0.5 (0.5–0.5) <0.001	0.3 (0.3–0.3) <0.001	0.5 (0.4–0.5) <0.001	0.7 (0.7–0.7) <0.001	-0.8 (-0.9–-0.8) <0.001	-0.0 (-0.0–0.0) 0.521
**Hyperlipidemia**	0.3 (0.3–0.3) <0.001	0.8 (0.8–0.8) <0.001	0.5 (0.5–0.5) <0.001	0.7 (0.7–0.8) <0.001	-0.8 (-0.8–-0.8) <0.001	0.2(0.2–0.2) <0.001
**Diabetes Mellitus**	0.9 (0.8–0.9) <0.001	2.0 (2.0–2.0) <0.001	1.0 (0.9–1.0) 0.080	0.7 (0.7–0.8) <0.001	0.4 (0.4–0.4) <0.001	0.1 (0.1–0.1) <0.001
**Congestive Heart Failure**	2.6 (2.5–2.7) <0.001	6.2 (6.0–6.3) <0.001	4.4 (4.3–4.5) <0.001	1.0 (1.0–1.1) 0.653	2.9 (2.8–2.9) <0.001	0.4 (0.4–0.4) <0.001
**Chronic Obstructive Pulmonary Disease**	1.3 (1.2–1.4) <0.001	1.3 (1.3–1.4) <0.001	1.7 (1.6–1.8) <0.001	0.9 (0.8–0.9) <0.001	0.6 (0.6–0.7) <0.001	0.1 (0.1–0.1) <0.001
**Weight loss**	1.1 (0.7–1.9) 0.613	1.3 (1.0–1.7) 0.048	0.9 (0.6–1.4) 0.929	0.8 (0.5–1.2) 0.280	0.7 (0.4–1.0) <0.001	0.1 (-0.1–0.3) 0.332
**Chronic Kidney Disease**	1.8 (1.7–1.9) <0.001	10.2 (10.0–10.5) <0.001	1.9 (1.8–1.9) <0.001	1.0 (1.0–1.1) 0.195	2.0 (2.0–2.1) <0.001	0.6 (0.6–0.6) <0.001
**Ischemic Stroke**	0.8 (0.7–0.9) <0.001	1.9 (1.8–2.0) <0.001	1.0 (0.9–1.1) <0.897	0.7 (0.6–0.7) <0.001	0.2 (0.2–0.3) <0.001	0.4 (0.3–0.4) <0.001
**AMI type (%)**						
**ST-segment Elevation Myocardial Infarction**	1.0	1.0	1.0	1.0	0	0
**Non–ST-segment Elevation Myocardial Infarction**	0.4 (0.4–0.4) <0.001	1.5 (1.5–1.5) <0.001	0.3 (0.3–0.3) <0.001	0.7 (0.7–0.8) <0.001	-0.0 (-0.1–-0.0) <0.001	-0.3 (-0.3–-0.3) <0.001

Supplementary Table S3 HIV status and in-hospital mortality and outcomes in AMI patients who underwent PCI in different subgroups

**Exposure**	**HIV status**	**In-hospital mortality** OR, (95% CI) *P*-value	**Acute Kidney Injury** OR. (95% CI) *P*-value	**Cardiogenic shock** OR, (95% CI) *P*-value	**Length of Hospital Stay** *β*, (95% CI) *P*-value	**Number of ICD-9-CM Procedures on this discharge** *β*, (95% CI) *P*-value
**Age<60**	**Asymptomatic HIV**	0.8 (0.3–2.0) 0.659	1.2 (0.9–1.7) 0.266	0.7 (0.4–1.2) 0.178	-0.2 (-0.4–0.0) 0.087	-0.1 (-0.0–0.3) 0.060
	**Symptomatic HIV**	7.8 (4.8–12.6) <0.001	1.4 (0.9 ‑ 2.4) 0.155	1.4 (0.8–2.5) 0.285	1.2 (0.8–1.6) <0.001	0.4 (0.1–0.7) 0.006
**Age≥60**	**Asymptomatic HIV**	0.2 (0.1–1.0) 0.046	0.6 (0.3–0.9) 0.019	0.2 (0.0–0.7) 0.015	-1.4 (-1.8–-0.9) <0.001	-0.6 (-0.8–-0.3) <0.001
	**Symptomatic HIV**	2.0 (1.5–2.5) <0.001	1.1 (0.9–1.3) 0.488	1.2 (0.9–1.6) 0.195	1.6 (1.3–1.8) <0.001	0.4 (0.3–0.6) <0.001
**Male**	**Asymptomatic HIV**	0.4 (0.2–0.9) 0.025	0.7 (0.5–1.0) 0.047	0.4 (0.2–0.8) 0.003	-0.7 (-0.9–-0.4) <0.001	-0.1 (-0.2–0.1) 0.399
	**Symptomatic HIV**	3.6 (2.7–4.7) <0.001	1.5 (1.2–1.9) <0.001	1.6 (1.2–2.1) 0.002	1.6 (1.4–1.9) <0.001	0.4 (0.2–0.5) <0.001
**Female**	**Asymptomatic HIV**	0.3 (0.0–2.1) 0.226	0.4 (0.2–1.0) 0.058	0.9 (0.3–2.3) 0.771	-0.7 (-1.4–0.1) 0.071	-0.1 (-0.6–0.3) 0.554
	**Symptomat**	2.0 (1.3–2.9)	1.0 (0.7–1.3)	0.9 (0.5–1.4)	1.8 (1.4–2.2)	0.6 (0.3–0.8)

	ic HIV	<0.001	0.813	0.616	<0.001	<0.001
**Race (%)**						
**Caucasian**	**Asymptomatic HIV**	0.5 (0.2–1.4) 0.184	0.7 (0.4–1.0) 0.077	0.6 (0.3–1.2) 0.177	-0.6 (-1.0–-0.2) 0.002	0.0 (-0.2–0.3) 0.792
	**Symptomatic HIV**	2.6 (2.1–3.4) <0.001	1.2 (1.0–1.5) 0.055	1.3 (1.0–1.7) 0.063	1.5 (1.3–1.7) <0.001	0.4 (0.3–0.5) <0.001
**Other**	**Asymptomatic HIV**	0.2 (0.1–0.8) 0.014	0.5 (0.4–0.7) <0.001	0.4 (0.2–0.8) 0.012	-1.1 (-1.5–-0.8) <0.001	-0.2 (-0.4–-0.1) 0.005
	**Symptomatic HIV**	4.1 (2.3–7.3) <0.001	2.1(1.4–3.1) 0.001	1.3 (0.6–2.8) 0.457	3.1 (2.5–3.7) <0.001	0.7 (0.4–1.0) <0.001
**Primary expected payer (%)**						
**Medicare**	**Asymptomatic HIV**	0.1 (0.0–0.4) 0.005	0.4 (0.2–0.6) <0.001	0.1 (0.0–0.4) 0.002	-1.5 (-1.9–-1.2) <0.001	0.0 (-0.1–0.2) 0.636
	**Symptomatic HIV**	1.9 (1.4–2.6) <0.001	1.2 (1.0–1.5) 0.085	1.3 (1.0–1.9) 0.091	1.4 (1.1–1.6) <0.001	0.3 (0.2–0.5) <0.001
**Medicaid**	**Asymptomatic HIV**	1.4 (0.3–5.5) 0.674	0.7 (0.3–1.7) 0.492	0.5 (0.2–1.7) 0.283	-0.9 (-1.7–-0.1) 0.028	-0.3 (-0.7–0.1) 0.187
	**Symptomatic HIV**	5.4 (1.9–15.0) 0.001	1.0 (0.3–2.7) 0.941	0.7 (0.2–2.8) 0.593	1.4 (0.3–2.5) 0.011	0.7 (0.1–1.3) 0.020
**Private Insurance**	**Asymptomatic HIV**	0.5 (0.1–3.8) 0.525	1.1 (0.5–2.2) 0.842	0.9 (0.3–2.3) 0.784	0.0 (-0.3–0.3) 0.968	-0.1 (-0.4–0.2) 0.481
	**Symptomatic HIV**	10.0 (6.3–15.9) <0.001	2.1 (1.3–3.4) 0.004	2.6 (1.5–4.5) 0.001	1.8 (1.5–2.1) <0.001	0.7 (0.5–1.0) <0.001
**Other**	**Asymptomatic HIV**	0.9 (0.3–2.8) 0.838	1.0 (0.6–1.7) 0.985	0.9 (0.4–1.8) 0.699	0.1 (-0.5–0.7) 0.758	-0.6 (-1.0–-0.1) 0.011
	**Symptomatic HIV**	2.0 (1.2–3.4) 0.012	0.8 (0.5–1.2) 0.301	0.8 (0.4–1.3) 0.313	2.0 (1.1–2.9) <0.001	0.3 (-0.3–0.9) 0.375
**Non-smoking**	**Asymptomatic HIV**	0.2 (0.1–0.8) 0.014	0.4 (0.3–0.7) <0.001	0.3 (0.1–0.7) 0.007	-1.2 (-1.6–-0.8) <0.001	0.2 (0.0–0.5) 0.025
	**Symptomatic HIV**	2.1 (1.6–2.8) <0.001	1.1 (0.8–1.3) 0.647	1.1 (0.8–1.5) 0.597	1.7 (1.4–2.0) <0.001	0.4 (0.3–0.6) <0.001
**smoking**	**Asymptomatic HIV**	0.6 (0.2–1.7) 0.345	1.2 (0.8–1.7) 0.450	0.7 (0.4–1.2) 0.166	-0.3 (-0.5–-0.0) 0.023	-0.3 (-0.5–-0.1) <0.001
	**Symptomatic HIV**	5.3 (3.7–7.6) <0.001	2.0 (1.5–2.6) <0.001	1.7 (1.2–2.5) 0.006	1.7 (1.4–1.9) <0.001	0.4 (0.2–0.6) <0.001
**Comorbidity (%)**						
**Non-Hypertension**	**Asymptomatic HIV**	0.3 (0.1–0.7) 0.009	0.5 (0.3–0.7) <0.001	0.3 (0.1–0.6) <0.001	-1.2 (-1.6–-0.8) <0.001	-0.4 (-0.6–-0.2) <0.001
	**Symptomatic HIV**	3.5 (2.6–4.7) <0.001	1.5 (1.2–1.9) <0.001	1.6 (1.1–2.2) 0.005	2.0 (1.6–2.4) <0.001	0.7 (0.5–0.9) <0.001
**Hypertension**	**Asymptomatic HIV**	0.4 (0.1–1.2) 0.108	0.8 (0.5–1.3) 0.385	0.8 (0.4–1.5) 0.395	-0.6 (-0.8–-0.3) <0.001	0.3 (0.1–0.5) 0.001
	**Symptomatic HIV**	2.2 (1.5–3.2) <0.001	1.1 (0.8–1.5) 0.675	1.1 (0.7–1.6) 0.794	1.4 (1.2–1.6) <0.001	0.2 (-0.0–0.3) 0.068
**Non-Hyperlipidemia**	**Asymptomatic HIV**	0.5 (0.2–1.1) 0.071	0.8 (0.5–1.2) 0.226	0.6 (0.3–1.1) 0.084	-0.8 (-1.3–-0.3) <0.001	-0.1 (-0.3–0.2) 0.558
	**Symptomatic HIV**	1.9 (1.5–2.5) <0.001	1.1 (0.8–1.3) 0.600	1.0 (0.8–1.4) 0.796	1.5 (1.2–1.9) <0.001	0.5 (0.4–0.7) <0.001
**Hyperlipidemia**	**Asymptomatic HIV**	0.2 (0.0–1.2) 0.072	0.6 (0.4–0.8) 0.003	0.4 (0.2–0.9) 0.024	-0.7 (-0.9–-0.5) <0.001	-0.1 (-0.3–0.1) 0.277
	**Symptomatic HIV**	5.1 (3.5–7.6) <0.001	1.6 (1.3–2.1) <0.001	1.6 (1.1–2.5) 0.023	1.7 (1.5–1.9) <0.001	0.3 (0.1–0.5) <0.001
**Non-Diabetes Mellitus**	**Asymptomatic HIV**	0.3 (0.1–0.8) 0.011	0.7 (0.5–1.0) 0.034	0.5 (0.3–0.9) 0.020	-0.6 (-0.9–-0.4) <0.001	-0.3 (-0.5–-0.1) <0.001
	**Symptomatic HIV**	3.2 (2.5–4.1) <0.001	1.7 (1.4–2.0) <0.001	1.3 (1.0–1.8) 0.041	2.0 (1.8–2.3) <0.001	0.6 (0.4–0.7) <0.001
	**Asymptom**	0.4 (0.1–1.4)	0.6 (0.4–0.9)	0.3 (0.1–0.9)	-1.2 (-1.6–-	0.5 (0.2–0.7)

**Diabetes Mellitus**	**atic HIV**	0.148	0.019	0.037	0.8) <0.001	<0.001
	**Symptomatic HIV**	1.5 (0.8–2.8) 0.153	0.9 (0.6–1.2) 0.414	1.2 (0.7–2.0) 0.517	1.0 (0.7–1.4) <0.001	0.1 (-0.1–0.3) 0.319
**Non-Congestive Heart Failure**	**Asymptomatic HIV**	0.2 (0.1–0.6) 0.005	0.9 (0.7–1.3) 0.625	0.4 (0.2–0.8) 0.014	-0.4 (-0.6–-0.3) <0.001	0.0 (-0.1–0.2) 0.813
	**Symptomatic HIV**	3.1 (2.3–4.1) <0.001	1.6 (1.3–2.0) <0.001	1.4 (1.0–2.0) 0.044	1.5 (1.3–1.7) <0.001	0.3 (0.2 – 0.5) <0.001
**Congestive Heart Failure**	**Asymptomatic HIV**	1.0 (0.4–2.9) 0.926	0.5 (0.3–0.9) 0.027	0.8 (0.4–1.8) 0.637	-1.9 (-3.1–-0.7) 0.003	-0.5 (-1.1–0.1) 0.092
	**Symptomatic HIV**	2.0 (1.4–3.0) <0.001	0.8 (0.6–1.0) 0.048	0.9 (0.6–1.3) 0.686	1.8 (1.1–2.4) <0.001	0.6 (0.3–0.9) <0.001
**Non-Chronic Obstructive Pulmonary Disease**	**Asymptomatic HIV**	0.3 (0.1–0.7) 0.004	0.6 (0.5–0.9) 0.003	0.5 (0.3–0.8) 0.005	-0.8 (-1.0–-0.6) <0.001	-0.1 (-0.2–0.1) 0.363
	**Symptomatic HIV**	3.2 (2.5–4.1) <0.001	1.6 (1.3–1.9) <0.001	1.4 (1.0–1.9) 0.027	1.6 (1.4–1.8) <0.001	0.5 (0.3–0.6) <0.001
**Chronic Obstructive Pulmonary Disease**	**Asymptomatic HIV**	0.7 (0.1–5.1) 0.734	0.5 (0.2–1.7) 0.307	0.3 (0.0–2.5) 0.285	-0.9 (-2.1–0.3) 0.123	0.0 (-0.7–0.7) 0.977
	**Symptomatic HIV**	1.7 (1.1–2.8) 0.016	0.6 (0.4–0.8) 0.005	0.8 (0.5–1.3) 0.324	1.9 (1.3–2.5) <0.001	0.2 (-0.1–0.6) 0.193
**Non-Chronic Kidney Disease**	**Asymptomatic HIV**	0.3 (0.1–0.7) 0.007	0.7 (0.5–0.9) 0.0201	0.5 (0.3–0.8) 0.005	-0.7 (-1.0–-0.5) <0.001	-0.0 (-0.2–0.1) 0.869
	**Symptomatic HIV**	2.9 (2.2–3.6) <0.001	1.2 (1.0–1.5) 0.083	1.3 (1.0–1.7) 0.055	1.6 (1.4–1.8) <0.001	0.4 (0.3–0.5) <0.001
**Chronic Kidney Disease**	**Asymptomatic HIV**	0.4 (0.0–2.6) 0.305	0.7 (0.4–1.1) 0.100	0.4 (0.1–1.7) 0.216	-1.0 (-2.3–0.2) 0.108	-0.5 (-1.1–0.1) 0.075
	**Symptomatic HIV**	2.7 (1.5–4.7) <0.001	1.5 (1.1–2.0) 0.016	1.3 (0.7–2.4) 0.440	1.7 (0.9–2.6) <0.001	0.4 (0.0–0.8) 0.032
**AMI TYPE (%)**						
**ST-segment Elevation Myocardial Infarction**	**Asymptomatic HIV**	0.5 (0.2–1.2) 0.109	1.3 (0.9–2.0) 0.182	0.7 (0.4–1.2) 0.206	-0.4 (-0.8–0.1) 0.098	-0.3 (-0.6–-0.0) 0.023
	**Symptomatic HIV**	2.9 (2.2–3.8) <0.001	1.5 (1.1–1.9) 0.003	1.3 (1.0–1.7) 0.099	1.5 (1.2–1.8) <0.001	0.5 (0.3–0.7) <0.001
**Non-ST-segment Elevation Myocardial Infarction**	**Asymptomatic HIV**	0.2 (0.1–0.9) 0.036	0.4 (0.3–0.6) <0.001	0.3 (0.1–0.8) 0.016	-1.0 (-1.3 – -0.7) <0.001	0.1 (-0.1–0.3) 0.241
	**Symptomatic HIV**	2.4 (1.6–3.6) <0.001	1.2 (1.0–1.5) 0.090	1.1 (0.7–1.9) 0.616	1.9 (1.6–2.1) <0.001	0.4 (0.2–0.5) <0.001

Supplementary Table S4 Baseline characteristics of AMI patients who underwent PCI grouped by HIV status after 1:2 case-control matching

Variable	HIV-negative (n=3,726)	HIV-positive (n=1,863)	*P*-value
**Age (Mean±SD)**	62.9±12.2	62.9±12.2	1.000
**Female (%)**	906 (24.3%)	453 (24.3%)	1.000
**Race (%)**			<0.001
**Caucasian**	2877 (77.2%)	1228 (65.9%)	
**African American**	263 (7.1%)	312 (16.7%)	
**Hispanic**	297 (8.0%)	256 (13.7%)	
**Asian or Pacific Islander**	125 (3.4%)	13 (0.7%)	
**Native American**	7 (0.2%)	4 (0.2%)	
**Other**	157 (4.2%)	50 (2.7%)	
**Primary expected payer (%)**			<0.001
**Medicare**	1642 (44.1%)	958 (51.4%)	
**Medicaid**	284 (7.6%)	161 (8.6%)	
**Private Insurance**	1491 (40.0%)	405 (21.7%)	
**Other**	309 (8.3%)	339 (18.2%)	
**Median household income (%)**			<0.001
**1st quartile**	860 (23.1%)	657 (35.3%)	
**2nd quartile**	976 (26.2%)	413 (22.2%)	
**3rd quartile**	948 (25.4%)	379 (20.3%)	
**4th quartile**	942 (25.3%)	414 (22.2%)	
**Smoking (%)**	1924 (51.6%)	962 (51.6%)	1.000
**Previous Coronary Artery Bypass Graft**	159 (4.3%)	62 (3.3%)	0.089
**Hypertension**	1778 (47.7%)	889 (47.7%)	1.000
**Hyperlipidemia**	1868 (50.1%)	934 (50.1%)	1.000
**Diabetes Mellitus**	832 (22.3%)	416 (22.3%)	1.000
**Congestive Heart Failure**	618 (16.6%)	309 (16.6%)	1.000
**Chronic Obstructive Pulmonary Disease**	368 (9.9%)	184 (9.9%)	1.000
**Weight Loss**	6 (0.2%)	11 (0.6%)	<0.001
**Chronic Kidney Disease**	386 (10.4%)	193 (10.4%)	1.000
**Ischemic Stroke**	62 (1.7%)	31 (1.7%)	1.000
**AMI type (%)**			1.000
**ST-segment Elevation Myocardial Infarction**	1562 (41.9%)	781 (41.9%)	
**Non–ST-segment Elevation Myocardial Infarction**	2164 (58.1%)	1082 (58.1%)	

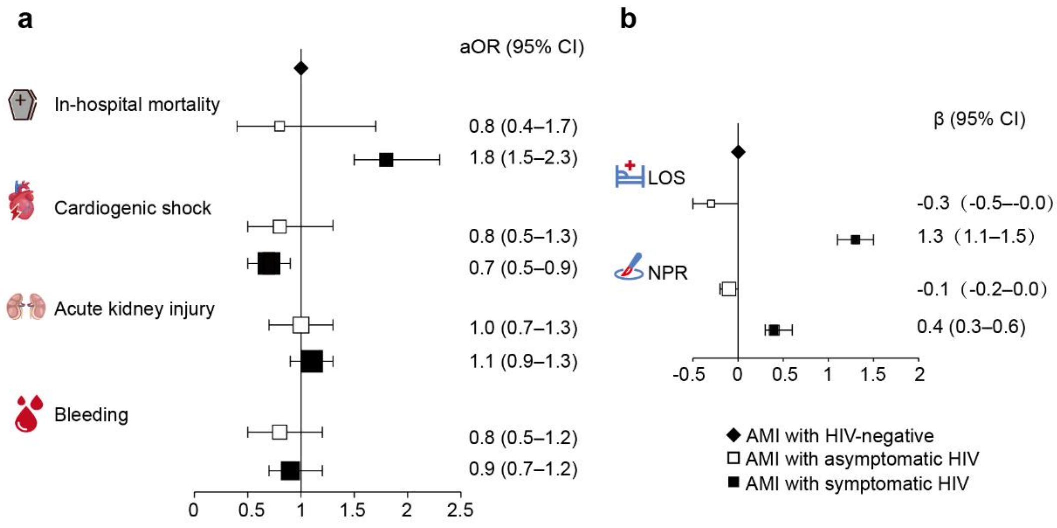

Supplementary Figure 1. In-hospital mortality and outcomes in non-matching model in AMI underwent PCI procedure Asymptomatic HIVAIDS and Symptomatic HIVAIDS cohorts

Figure legends:

Supplementary Figure 1. In-hospital mortality and outcomes in non-matching model. (A) Adjusted odd ratios for in-hospital mortality and outcomes. (B) Adjusted β value for length of stay (LOS) and additional procedures during hospitalization (NPR); represented as odds ratio and β value (95% CI). Adjusted for age, sex, race, primary payer, socioeconomic status, smoking, prior coronary artery bypass graft, and comorbidity.
